# A case of subcutaneous sarcoidosis in a patient with Cushing's syndrome^⋆^

**DOI:** 10.1016/j.abd.2021.05.018

**Published:** 2022-05-25

**Authors:** Kinuko Irie, Toshiyuki Yamamoto

**Affiliations:** Department of Dermatology, Fukushima Medical University, Fukushima, Japan

**Keywords:** Cushing syndrome, Glucocorticoids, Sarcoidosis

## Abstract

A 41-year-old female visited our department complaining of asymptomatic subcutaneous nodules on the right forearm. She had been diagnosed as having Cushing syndrome due to an adrenal tumor 5-months previously. After she underwent surgery for the adrenal tumor, the subcutaneous nodules gradually increased in number. Physical examination showed ill-defined plate-like subcutaneous indurations on the bilateral lower extremities, buttocks, and right forearm. A biopsy of one of the subcutaneous indurations showed non-caseating epithelioid cell granulomas involving the hypodermis and subcutaneous tissues. The patient was diagnosed as having sarcoidosis based on the Japan Society of Sarcoidosis and Other Granulomatous Disorders 2015 criteria. Skin lesions decreased in size and had completely disappeared. Although the mechanism is unknown, there may be a possibility that the activity of sarcoidosis is suppressed by high cortisol concentrations due to Cushing syndrome.

## Case report

There have been many immune-related diseases that have newly developed in patients with ' 'Cushing's syndrome following treatment, including arthritis, vasculitis, celiac disease, systemic lupus erythematosus, and sarcoidosis. The authors here report a case of sarcoidosis with ' 'Cushing's syndrome, along with the relevant literature.

**Figure 1:**
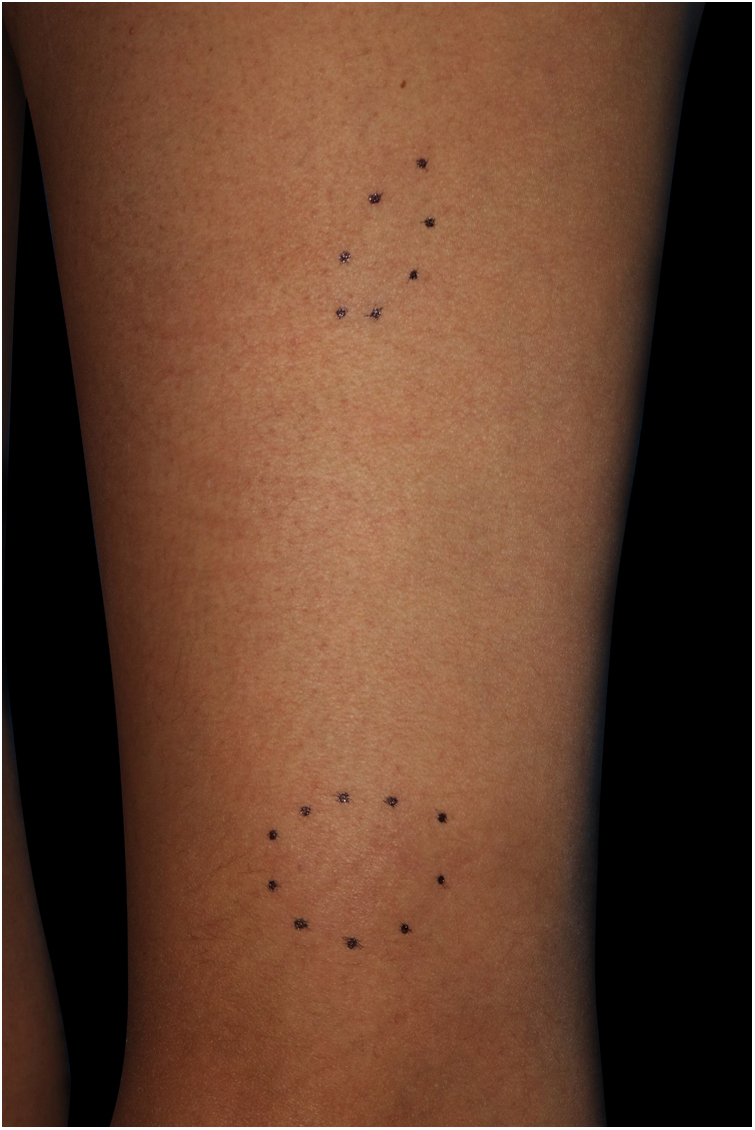
Subcutaneous nodules on the left thigh.

**Figure 2:**
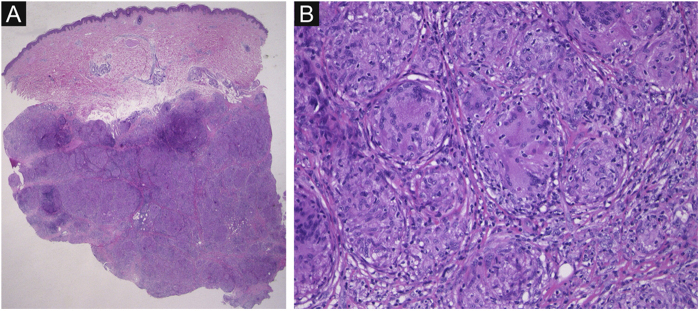
(A), Histological features showing dense localization of non-necrotizing epithelioid granulomas in the subcutaneous tissues. (Hematoxylin & eosin, ×12.5). (B), Higher magnification revealing granulomas with giant cells. (Hematoxilyn & eosin, ×200).

A 41-year-old female (Fitzpatrick skin type III) visited our department complaining of asymptomatic subcutaneous nodules on the right forearm. She had familial hypertension and had been diagnosed as having ' 'Cushing's syndrome due to an adrenal tumor 5-months previously. Around that time, she first noticed the subcutaneous nodules. Although she was taking oral hydrocortisone at a dose of 10 mg/day, after she underwent surgery for the adrenal tumor, the subcutaneous nodules gradually increased in number. Physical examination showed ill-defined plate-like subcutaneous indurations on the bilateral lower extremities, buttocks, and right forearm (Fig. 1). There was no palpable lymphadenopathy. There is a biopsy of one of the subcutaneous indurations that showed non-caseating epithelioid cell granulomas involving the subcutaneous tissues (Fig. 2). Chest X-Ray showed bilateral hilar lymphadenopathy, and computed tomography revealed mediastinal lymphadenopathy. Laboratory examination showed normal liver and renal function, as well as increased levels of angiotensin-converting enzyme (38.2 IU/L normal; 7–25) and soluble interleukin-2 receptor (937 U/mL, normal; 121–613). Although further examination excluded both cardiac and ocular sarcoidosis, the patient was diagnosed as having sarcoidosis based on the Japan Society of Sarcoidosis and Other Granulomatous Disorders 2015 criteria. The skin lesions were followed-up with monitoring and continuous administration of oral hydrocortisone each day, the dose of which was tapered over a period of 6-months following the surgery. During the same period, the skin lesions decreased in size and had completely disappeared at 20-months after the surgery.

## Discussion

There have been many immune-related diseases that have newly developed in patients with ' 'Cushing's syndrome following treatment, including arthritis, vasculitis, celiac disease, systemic lupus erythematosus, and sarcoidosis.^1^ To date, some cases of co-existing sarcoidosis and ' 'Cushing's syndrome, with cutaneous sarcoidosis have been reported. Subcutaneous sarcoidosis is reported to occur in 1.4%–6% of patients with systemic sarcoidosis. Several cases of sarcoidosis developing after treatment for ' 'Cushing's syndrome have been reported;^1^, ^2^, ^3^, ^4^, ^5^, ^6^, ^7^, ^8^, ^9^, ^10^ however, the patient in the current report noticed subcutaneous nodules on her forearm around the same time as when she was diagnosed as having ' 'Cushing's syndrome.

In the literature review, the authors found 10 case reports on PubMed of sarcoidosis after treatment for ' 'Cushing's syndrome between 1967 and 2020. The authors used the term """Cushing""" and """sarcoidosis""". The clinical features from those case reports^1^, ^2^, ^3^, ^4^, ^5^, ^6^, ^7^, ^8^, ^9^, ^10^ and the current case are given in Table 1. The previous case reports included one male and 9 females with a median age of 37 years (range, 27–45). The diagnosis of ' 'Cushing's syndrome was made in all cases. The mean time to onset of sarcoidosis symptoms after treatment of ' 'Cushing's syndrome was 5-months (range, 1–72). All patients had subcutaneous nodules as sarcoidosis symptoms. Two patients also had erythema nodosum, indicating sarcoidosis. Nine patients had pulmonary complications, and only one patient had ocular complications. No patients had cardiac complications, but all patients had skin symptoms. After treatment for ' 'Cushing's syndrome, subcutaneous sarcoidosis was more frequently reported than any other form of cutaneous sarcoidosis.

**Table 1:** The clinical features of similar reports and the current case.

Authors	Gender	Age	Underlying disease	Involved organs	Latency (months)	Clinical features
Bongetta et al.^1^	F	33	PA	Lungs and skin	12	Subcutaneous nodules
Noreña et al.^2^	F	45	AA	Lungs and skin	5	Hypercalcemia, arthralgia, subcutaneous nodules
Fichtel et al.^3^	F	42	PA	Skin	3	Subcutaneous nodules, pink hypertrophic scars
Diernaes et al.^4^	F	45	PA	Lungs and skin	3	Erythema nodusum, painful subcutaneous nodules
Takenaka et al.^5^	F	32	PA	Lungs, eyes, and skin	72	Erythema nodosum, subcutaneous nodules, granulomatous uveitis
Tanaka et al.^6^	F	37	AA	Lungs and skin	24	Subcutaneous nodules
Steuer et al.^7^	F	42	AA	Lungs and skin	1.5	Subcutaneous nodules
Schaefer et al.^8^	F	32	AA	Lungs and skin	1	Subcutaneous nodules, stiffness, and arthralgia
Marzano et al.^9^	F	33	PA	Lungs and skin	2	Subcutaneous nodules
da Mota et al.^10^	M	27	PA	Lungs and skin	12	Subcutaneous nodules
Current case	F	41	AA	Lungs and skin	1	Subcutaneous nodules

F, Female; M, Male; PA, Pituitary Adenoma; AA, Adrenal Adenoma.

Although the mechanism is unknown, there may be a possibility that the activity of sarcoidosis is suppressed by high concentrations of cortisol due to ' 'Cushing's syndrome. In the current case, perhaps the improvement of cortisol concentration after surgery for an adrenal tumor may have resulted in a more clearly identifiable case of sarcoidosis. Patients with ' 'Cushing's syndrome need to be carefully followed up after treatment because they can develop sarcoidosis, which may be overlooked in the absence of the characteristic skin lesions.

## Financial support

None declared.

## Authors' contributions

Kinuko Irie: Designed the study; performed the research and contributed to the analysis and interpretation of data; wrote the initial draft of the manuscript; read and approved the final version of the manuscript.

Toshiyuki Yamamoto: Designed the study; assisted in the preparation of the manuscript; read and approved the final version of the manuscript.

## Conflicts of interest

None declared.
